# Conservative surgical management of surface osteosarcoma using 3D printing technology: An unusual case report and literature review

**DOI:** 10.1016/j.ijscr.2023.109086

**Published:** 2023-11-24

**Authors:** Mohamed Amine Gharbi, Ahmed Zendeoui, Anis Tborbi, Ramzi Bouzidi, Khelil Ezzaouia, Mouadh Nefiss

**Affiliations:** Department of Orthopedic and Trauma Surgery, Mongi Slim Marsa University Hospital Center, Tunis, Tunisia

**Keywords:** Surface osteosarcoma, Conservative treatment, 3D printing technology, Case report

## Abstract

**Introduction and importance:**

Surface osteosarcoma, a rare variant of osteosarcoma, poses unique challenges in diagnosis and treatment. This report discusses the application of 3D printing technology in the surgical management of a complex case involving a 27-year-old female patient with surface osteosarcoma in the proximal tibia.

**Case presentation:**

A 27-year-old female patient presented with a progressively growing mass on her right knee, initially misdiagnosed as a benign bone tumor. Over five years, the lesion expanded from a well-corticated metaphyseal-epiphyseal outgrowth on the proximal tibia to involve the anterior tibial tuberosity. Radiological and histological evaluations confirmed well-differentiated paraosteal surface osteosarcoma. A multidisciplinary team opted for a conservative surgical approach, including resection of the anterior tibial tuberosity and patellar tendon. Precision was enhanced through 3D printing technology, which provided custom cutting guides. The reconstruction involved non-vascularized peroneus and iliac crest bone grafts.

**Clinical discussion:**

Accurate differentiation from benign lesions presents challenges. Achieving surgical resection with clear margins is pivotal for favourable outcomes, particularly in young patients. Chemotherapy yields limited benefits in low-grade surface osteosarcomas. Functional prognosis hinges on effective post-resection reconstruction. 3D printing technology facilitates meticulous surgical planning and guidance, enhancing the success of conservative surgical interventions.

**Conclusion:**

This case underscores the significance of a multidisciplinary approach, accurate diagnosis, and the integration of 3D printing technology in managing surface osteosarcomas. Conservative surgical resection, guided by precise planning and reconstruction, is critical for preserving functionality. Continued research and the adoption of innovative techniques hold promise for improving the quality of life and functional outcomes of individuals grappling with musculoskeletal tumors.

## Highlights

• Surface osteosarcoma challenges: diagnosis, surgical management, vital and functional prognosis.
• Conservative resection with clear margins is crucial for favourable outcomes.
• Pre-operative planning is primordial to succeed safe margins surgical resection and appropriate reconstruction.
• 3D printing technology aids precise surgical planning for complex bone tumors.
• Multidisciplinary approach enhances musculoskeletal tumor management.

## 1 Introduction

Surface osteosarcoma, is a subtype of osteosarcoma, which is a rare and aggressive form of bone cancer. Unlike conventional osteosarcoma, which typically arises within the medullary cavity of long bones, surface osteosarcoma originates on the surface of bones, particularly in the extremities [1]. It tends to develop near the cortex of the bone and is often associated with the outer lining of the periosteum.

Surface osteosarcoma is known for its relatively slow growth compared to intramedullary osteosarcoma [2]. However, it can still exhibit local aggressiveness and the potential to metastasize to the lungs [3]. It primarily affects young adults and poses a dual diagnostic challenge, both in terms of imaging and histological examination, as it can be easily confused with several differential diagnoses [4]. Moreover, the therapeutic aspect is complex, especially due to its typical location near joints in the metaphyseal-epiphyseal region, which emphasizes the importance of conservative treatment in young patients. Another crucial aspect of its management is the reconstruction following resection, which significantly influences functional prognosis and requires meticulous preoperative planning.

Hereby, a case of a slowly progressing paraosteal osteosarcoma of the proximal tibia, involving the tibial tuberosity with a posterior-medial extension that posed challenges for resection and reconstruction. We highlight how the initial diagnosis was mistakenly considered as benign bone tumor without any indications of malignancy. Furthermore, our research delves into the utilization of 3D printing within our orthopedic surgery department for treatment planning and intraoperative guidance, using a specific cutting guide that ensured a surgically sound resection with clear margins. Such a resection is the only guarantee of effective surgical management without the risk of local recurrence.

This case report has been reported in line with the SCARE criteria [5].

## 2 Case presentation

**Fig. 1:**
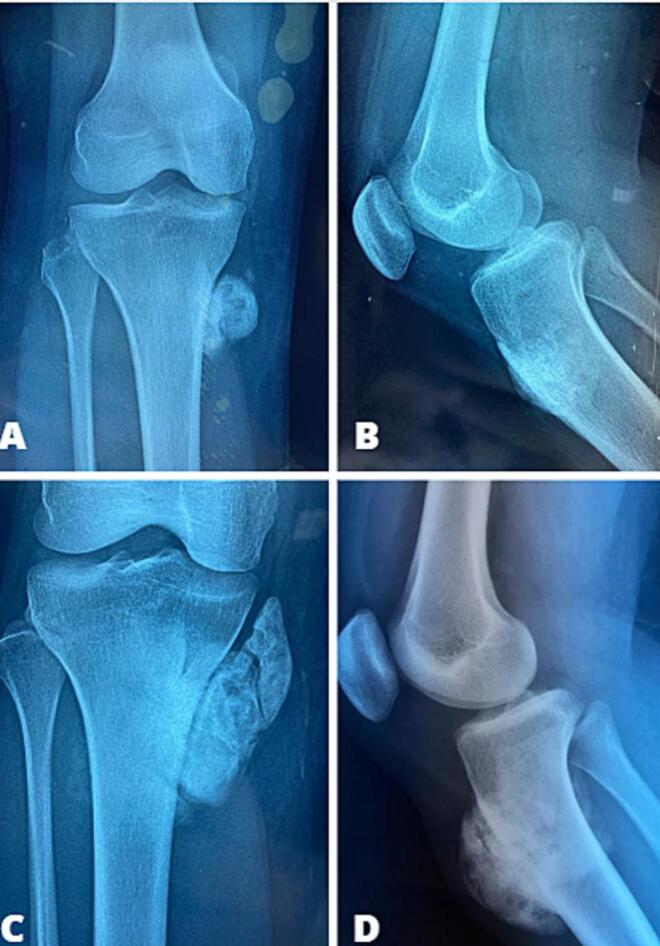
The radiological aspect of a metaphyseal osteosclerotic mass measuring 4 cm on images A and B, which has increased in size after 5 years (C and D).

We report the case of a 27-year-old female patient with no significant medical history, who presented with mixed-type right knee pain related to a swelling on the anteromedial aspect of the knee. This swelling had been progressively developing over the past 5 years, with no signs of local inflammation or limitation of mobility. A standard knee X-ray performed showed a well-corticated, homogeneously structured metaphyseal-epiphyseal bony outgrowth on the proximal tibia, measuring 4 cm along its major axis, with no cortical breach or periosteal reaction (Fig. 1).

**Fig. 2:**
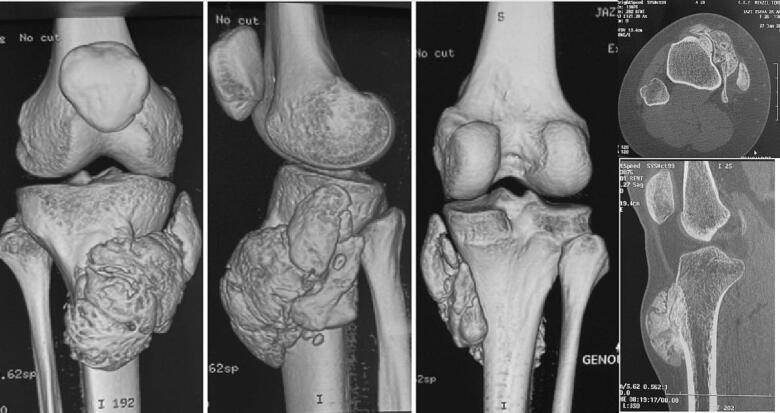
CT scans showing a tumor involving the cortical region without medullary extension.

**Fig. 3:**
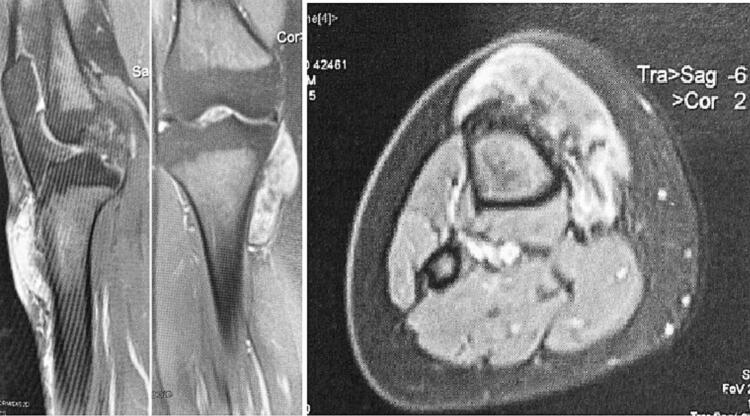
MRI images demonstrating invasion of the anterior tibial tuberosity and the patellar tendon without extension to the posterior and external soft tissues of the leg.

The initial radiological diagnosis was a benign bone tumor (osteochondroma), and the patient did not follow up. She presented to us after 5 years with an enlarged mass that had not affected knee mobility. There was no sensory-motor deficit, and no systemic symptoms were reported. A subsequent X-ray revealed an increased size of the lesion, measuring 7 cm along its major axis involving the anterior tibial tuberosity (Fig. 1). Additional CT and MRI scans indicated no medullary extension, and regional staging did not reveal distant metastases (Fig. 2, Fig. 3). A surgical biopsy was performed, confirming the diagnosis of well-differentiated parosteal surface osteosarcoma.

**Fig. 4:**
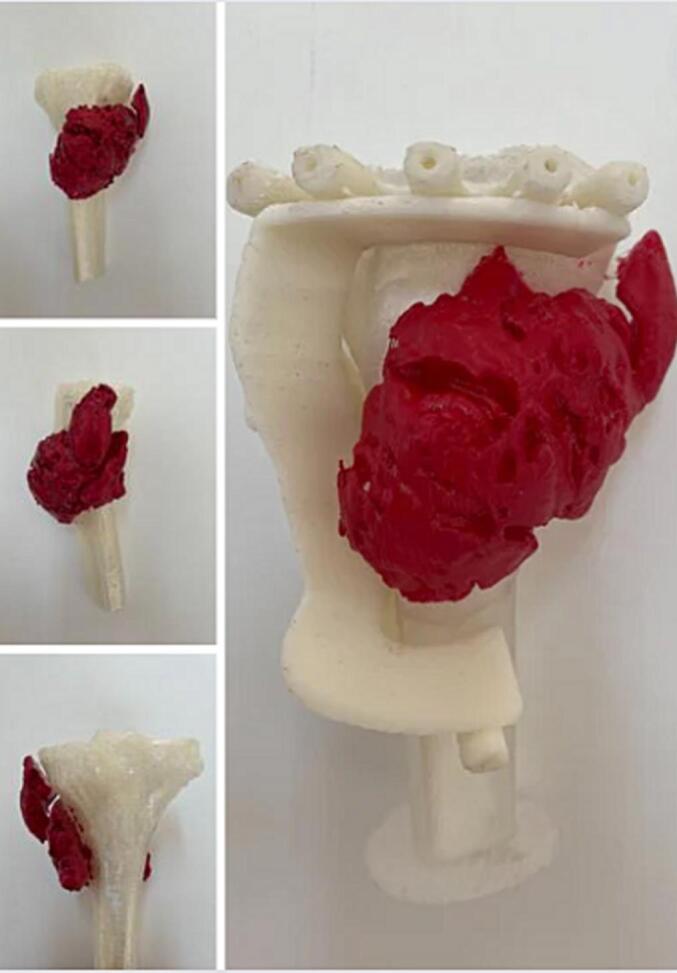
3D printing of the proximal tibia with the presence of the tumor, along with the custom-made cutting guide well adapted to the bony contours of the proximal end of the tibia and is anchored in place with pins.

A multidisciplinary team decision was made to perform a conservative oncological surgical resection of the knee joint, including the involved anterior tibial tuberosity and patellar tendon. Preoperative planning for resection and reconstruction using 3D printing technology to enable precise complete resection with the assistance of a customized cutting guide. Anticipating the expected bone loss and accurately calculated dimensions of a thin posterior-lateral cortical defect (Fig. 4).

The surgical procedure involved resection of the tumor, which encompassed the anterior tibial tuberosity (TTA), half of the patellar tendon, the pes anserinus, and the superficial bundle of the lateral collateral ligament (LCL), while preserving the deep bundle of the LCL. There was no need for opening the joint capsule.

In terms of the reconstruction procedure, we used the non-vascularized peroneus for the posterior-medial pillar, embedded within the tibial shaft. The iliac crest bone graft was employed for the anterior tibial tuberosity and for the anterior-medial defect. The patellar tendon was reconstructed using the long peroneal tendon. Finally, we reinforced the posterior-internal structure through transosseous reinsertion of the hamstring muscles tendons following osteosynthesis with a locking screw plate.

**Fig. 5:**
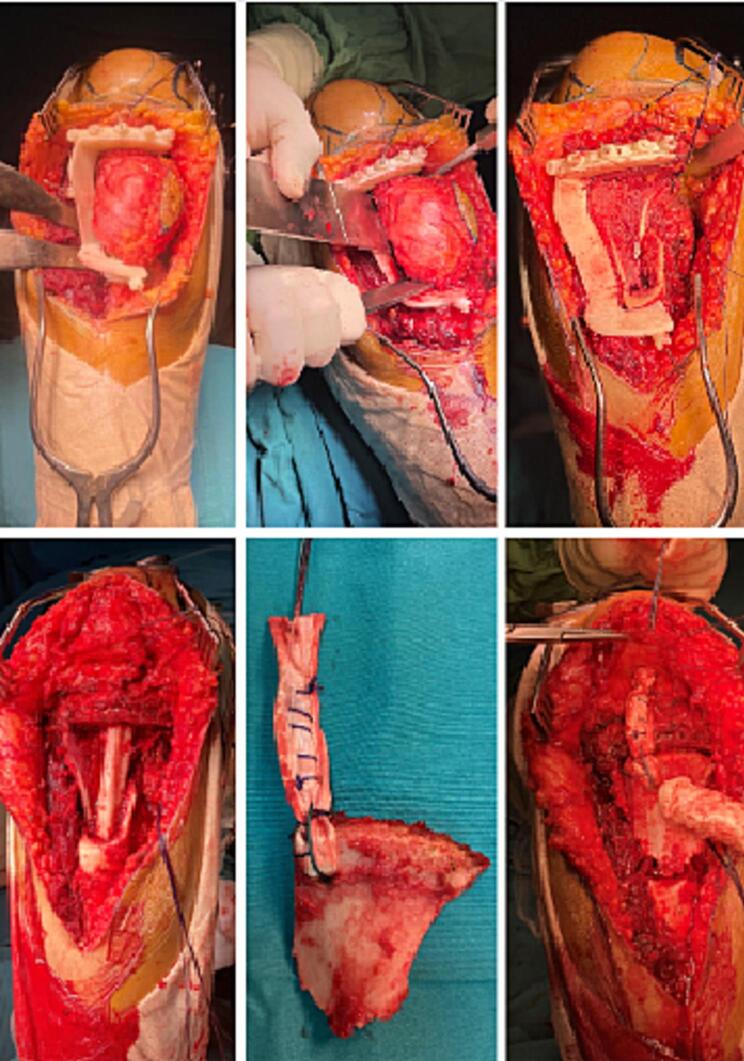
Intraoperative use of the cutting guide for tumor resection and reconstruction using a peroneal graft and the iliac crest with the long peroneal ligament for the extensor apparatus.

**Fig. 6:**
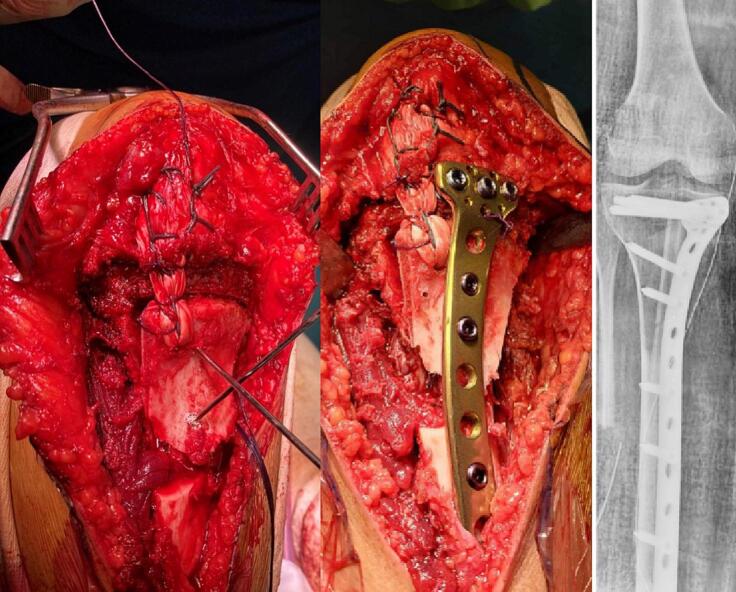
The final appearance after tumor resection and reconstruction.

Postoperatively, the patient was immobilized with a removable knee brace and instructed to avoid weight-bearing for 6 weeks (Fig. 5, Fig. 6). Subsequently, a gentle rehabilitation program was initiated. The patient retained a competent extensor system but had slight knee stiffness with a flexion deficit of 5 degrees.

**Fig. 7:**
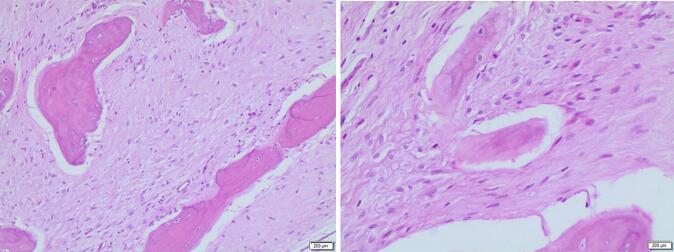
Microscopic appearance confirming the diagnosis of low grade paraosteal osteosarcoma.

Histopathological examination of the resected specimen confirmed low grade paraosteal osteosarcoma with clear resection margins (Fig. 7). Over the past eighteen months of follow-up, no signs of recurrence were observed, and positive functional outcomes were noted in the lower limb, and the patient regained satisfactory native knee function.

## 3 Discussion

Surface osteosarcoma is subdivided into three subgroups with varying incidence rates: low-grade osteosarcomas, including parosteal osteosarcoma with an incidence of 5 % of all osteosarcomas, and periosteal osteosarcoma with an incidence of 1.5 %. On the other hand, high-grade surface osteosarcoma has a lower incidence rate of 0.5 % [6].

Surface osteosarcomas typically affect individuals in the young to middle-aged adult population. While mean and median ages are commonly reported to be in the 20s, it's worth noting that the age range can vary widely, spanning from 8 to 85 years [7,8].

Surface osteosarcomas are tumors that develop from the outer cortical and periosteal layers of the bone, setting them apart from conventional osteosarcomas that originate within the bone's medullary cavity.

There are two primary subtypes: Low-Grade Surface Osteosarcoma with a less aggressive behavior and a lower likelihood of metastasis, and the High-Grade Surface Osteosarcoma, which is -In contrast- more aggressive and has a greater potential for metastasis with a higher risk of recurrence after treatment; A more aggressive approach, such as wide surgical excision and possibly adjuvant chemotherapy, may be necessary.

In the literature, a classification system has been proposed to distinguish between parosteal and periosteal osteosarcomas and provide treatment guidelines. It identifies low-grade, slow-growing forms suitable for surgical intervention (e.g., parosteal and periosteal osteosarcomas) and high-grade forms requiring a combination of chemotherapy and surgery due to their aggressive potential (e.g., ‘dedifferentiated’ parosteal osteosarcoma and high-grade surface osteosarcoma) [9].

In low-grade lesions, particularly in cases of classic parosteal osteosarcoma, symptoms tend to persist over an extended period; as seen in our case where symptoms had been progressing for five years. Conversely, in high-grade secondary osteosarcoma and dedifferentiated primary osteosarcoma, particularly when dedifferentiation occurs concurrently, the patient's clinical history is indistinguishable from that of typical osteosarcoma [10].

Periosteal osteosarcomas commonly manifest in younger patients and typically occur in the diaphyseal region [11]. They are characterized by a predominantly chondroid matrix. Radiographically, they exhibit a distinctive pattern where they radiate perpendicular to the bone cortex, resulting in cortical scalloping. Unlike parosteal osteosarcomas, periosteal osteosarcomas do not typically display the dense osteoid matrix but often exhibit less dense chondroid calcification. On the other hand, high-grade surface osteosarcomas tend to be located in the diaphyseal region, and radiographically, they appear less mineralized. They also exhibit a growth pattern perpendicular to the long axis of the bone [12].

We also illustrate the difficulty of radiological diagnosis: it is a differential diagnosis of osteochondroma (a benign tumor with a risk of degeneration), but its radiological characteristics are different. It is a metaphyseal tumor that develops on the opposite side of the joint line with continuity with the bone marrow and cortex.

In summary, radiographic and CT imaging findings indicate that low-grade parosteal osteosarcomas are characterized by a dense osteoid matrix. MRI imaging further underscores this characteristic, revealing low-signal-intensity lesions on both T1- and T2-weighted images.

Bertoni et al. believed that the identification of a deep radiolucent area on a computed tomographic scan or other preoperative radiographic staging studies should raise concerns about the possible presence of a high-grade (grade-II) dedifferentiated parosteal osteosarcoma.

The histological diagnosis itself can also be confusing with other differential diagnoses, such as fibrous dysplasia, as was initially the case in the patient we presented. Therefore, we emphasize the importance of the complementarity and concordance between radiological and histological findings in the diagnostic process, which is achieved through multidisciplinary consultation meetings.

The surgical management of these surface osteosarcomas involves surgical resection that removes the cortical bone on which the parosteal osteosarcoma has developed when the medullary canal is not invaded. This procedure is considered safe and does not necessitate extensive resection. Intraluminal resection is strongly discouraged due to its significant association with recurrence and dedifferentiation [8]. This was performed in our case with replacement of the bone defect using two types of grafts, the fibula serving as support for the posterior column and the iliac crest fixed with a screw plate for the medial column. The most crucial aspect in the management of such tumors, especially in young patients, is conservative treatment while maintaining an oncological approach to ensure that it does not impact the functional prognosis of the affected limb.

Chemotherapy offers limited advantages in the treatment of surface osteosarcoma. Rose's study [13] demonstrated that chemotherapy, specifically using doxorubicin, ifosfamide, and methotrexate, did not provide any significant benefits in patients with PerOS. The level of necrosis observed in patients who received this chemotherapy regimen was comparable to the natural necrosis observed in resected specimens from other patients.

The prognosis of this tumor depends on the histological differentiation grade, the degree of invasion into the cortex, the treatment approach, the nature of surgical resection, and the margins of tumor resection [14]. A wide resection with histologically clear margins ensures a favourable long-term prognosis without recurrence [15].

The functional prognosis, on the other hand, depends on the reconstruction strategy following tumor resection in the case of conservative treatment. This reconstruction is often quite challenging. We emphasize here the importance of using modern technologies in preoperative planning, such as 3D reconstruction and printing of specific cutting guides.

Our case has shed light on the significance of meticulous therapeutic planning in the context of surface parosteal osteosarcoma. Through our case study, we have demonstrated the remarkable potential of personalized 3D-printed cutting guides in preserving the knee joint while effectively addressing bone replacement using peroneal and iliac bone grafts. Moreover, the restoration of extensor apparatus function through the use of the peroneal long tendon adds another dimension to the success of our approach.

Based on this article, we emphasize the value and importance of navigation and the use of 3D printing techniques in the conservative treatment and limb salvage of peripheral tumors. Its application is not limited to complex and hard-to-reach tumors of the pelvis and spine, as most publications in the literature tend to focus on; Moreover, this technique, allowing for a conservative approach, spares us from the high costs of personalized tumor-constrained prostheses. To our knowledge, we have found only one Chinese study from 2021 that demonstrated the use of 3D-printed cutting guides for planning tumor resection and creating custom implants. The cases reported in the study involved a high-grade osteosarcoma and an Ewing's sarcoma [16]. There were no illustrated cases specifically addressing surface osteosarcomas.

## 4 Conclusion

Through this illustrated case, we emphasize the importance of a multidisciplinary approach, preoperative planning utilizing new technologies such as 3D printing, in collaboration with healthcare technology specialists and engineers who can contribute to the management of complex cases by providing their specialized input to assist the surgeon in therapeutic decision-making and better patient care. This safe and cost-effective technology should be increasingly utilized in musculoskeletal tumor pathology.

Looking ahead, continued research and the adoption of such innovative techniques hold the promise of further improving the quality of life and functional outcomes for patients facing musculoskeletal tumors.

## Consent statement

Written informed consent was obtained from the patient for publication of this case report and accompanying images. A copy of the written consent is available for review by the Editor-in-Chief of this journal on request.

## Provenance and peer review

Not commissioned, externally peer-reviewed.

## Ethical approval

Ethical approval for this study was provided by the Ethical Committee of Mongi Slim University Hospitals, Marsa, Tunisia on 01 October 2023.

## Funding

This research did not receive any specific grant from funding agencies in the public, commercial, or not-for-profit sectors.

## Author contribution

Mohamed Amine Gharbi and Ahmed Zendeoui: wrote the manuscript and performed bibliographic research. Anis Teborbi and Mouadh Nefiss: contributed to the data analysis and collected iconographic elements. Ramzi Bouzidi and Khelil Ezzaouia: critically revised the manuscript and gave final approval.

## Guarantor

Ahmed Zendeoui.

## Research registration number

N/A.

## Conflict of interest statement

The author(s) declared no potential conflicts of interest.
